# The Impact of Peer Attachment on Left-Behind Children’s Pathological Internet Use: A Moderated Mediating Effect Model

**DOI:** 10.3390/ijerph18189775

**Published:** 2021-09-16

**Authors:** Demei Zhang, Shutao Wang

**Affiliations:** College of Education, Zhejiang University, Hangzhou 310058, China; 11903002@zju.edu.cn

**Keywords:** perceived personal rejection, emotional intelligence, peer attachment, pathological Internet use, left-behind children

## Abstract

The aim of this study was to determine how left-behind children’s perceived personal rejection and emotional intelligence impact on the relationship between their peer attachment and pathological Internet use in China. Using the cluster random sampling method, a total of 406 left-behind children (aging 12.76 ± 2.13) from six rural primary and secondary schools in mainland China were recruited for the study (202 males and 204 females). The results of the analysis indicated that peer attachment negatively predicted left-behind children’s pathological Internet use. Perceived personal rejection had a mediating effect on the relationship between peer attachment and pathological Internet use, whereas emotional intelligence had a moderating effect on the relationships between peer attachment and perceived personal rejection and between peer attachment and pathological Internet use among these children. Moreover, peer attachment had a greater negative impact on the perceived personal rejection and pathological Internet use of left-behind children with high emotional intelligence compared with those of students with low emotional intelligence. These findings reveal the need for more support and interventions aimed at strengthening peer attachment and emotional intelligence of left-behind primary and secondary children and reducing their perceptions of personal rejection, which, in turn, reduces their pathological Internet use.

## 1. Introduction

Pathological Internet use (PIU) among adolescents has attracted attention worldwide [1] and it refers to excessive use of the Internet, which damages individuals’ social and psychological functions and affects their normal work and learning outcomes [2]. Given its prevalence, the Internet plays an increasingly important role in the lives of children and teens. However, although the Internet greatly facilitates individuals’ work, study, and life activities, PIU has emerged as a serious problem [3]. An average global prevalence of Internet addiction has been estimated at around 6%; however, the detection rate of PIU among adolescents was 13.62% [4]. As more children overuse the Internet, their problem behaviors related to PIU have arisen accordingly. Studies indicate that excessive use of the Internet is more likely to lead to sleep-related problems [5], emotional issues, poor academic performance [6], and various physical and mental health problems.

“Left-behind children” refers to children who remain behind in their rural hometowns to complete their school education because one or both of their parents have left to work in urban areas, and they are separated from their parents for a duration of at least six months. Some studies have shown that, given a lack of parental supervision, left-behind children are more likely to attempt to escape from reality by seeking a virtual world through the Internet compared with their peers [7], and they are also more vulnerable to PIU [8]. The rapid growth in the number of left-behind children in China, the Philippines, Ecuador, South Africa, and other countries or regions in the world has attracted increasing attention [9], and specifically, PIU among this group is arousing considerable concern [10]. Studies have indicated that left-behind children are more prone to PIU than other children [11,12]. Although numerous studies have been conducted on factors influencing PIU, research on its influencing mechanisms is still at an early stage, resulting in a limited understanding of its etiology and mechanisms [13]. The theory of developmental psychopathology provides an explanatory framework for this influence mechanism. The theory focuses on the risk and protection factors of individuals in the face of adversity. Environment is an important element of risk, which constitutes the conditional factors for individuals to face the pressure of crisis through influencing individual factors. Risk effect is regulated by protection factors, including various internal qualities of individuals (such as emotional intelligence). The main point of this theory is that individuals with high protection factors are more likely to actively face risks and form appropriate resilience [14]. Therefore, using developmental psychopathology [14] as our theoretical framework, this study aimed at revealing the key influencing factors and mechanisms of PIU among left-behind children and identifying strategies to reduce their PIU.

### 1.1. Peer Attachment and Pathological Internet Use among Left-Behind Children

According to statistics released by the China Women’s Federation, in 2013, the number of left-behind children in China exceeded 61 million, and an annually rising trend is evident [15]. Left-behind children who are separated from their parents for long periods may rely on their peers to fulfil their critical needs for social interaction, support, a sense of security, and even for their positive development [16]. Therefore, among the many factors that affect the PIU of left-behind children, peer attachment is a key factor that should be explored [11]. Peer attachment refers to the intimacy established between adolescents and the relationship through which they provide each other with warmth and support [17]. It is a crucial form of attachment among children [18]. A supportive and close relationship with a peer enables an individual to adjust to their circumstances; in its absence, emotional and behavioral maladjustment could arise [19].

The relationship between peer attachment and PIU has been widely confirmed [20,21,22]. For example, one study conducted with secondary school students found that peer attachment could predict Internet addiction [23]. A second longitudinal study found that a close relationship exists between a lack of peer attachment and Internet gaming disorder [24]. According to attachment theory [25], a lack of attachment can lead to individuals feeling uncared for or unloved, which further impairs their social and psychological functions and contributes to their mental health problems. PIU is considered a common disorder and is included in the Diagnostic and Statistical Manual of Mental Disorders (DSM-V) [1]. Studies have also indicated that the risk of engaging in PIU is lower among adolescents who have a high level of peer attachment and high-quality relationships with their peers than among those who lack these relationships [21]. Adolescents with low levels of peer attachment are more likely to rely on the Internet (especially online games) for entertainment and social interaction and to over-engage with the Internet, leading to PIU compared with those with high levels of peer attachment [26].

Based on the above studies, the first hypothesis of this study is as follows:

**Hypothesis** **1** **(H1).** *Peer attachment is negatively related to pathological Internet use among left-behind children.*

### 1.2. Perceived Personal Rejection as a Mediator

There is increasing evidence that peer attachment does not have a direct impact on PIU; rather, it is mediated by various factors [22,26]. Behavior synthesis theory posits that children’s problem behaviors result from interactions of the social environment and individual factors [27]. Some studies have shown that peer attachment impacts on individuals’ mental health and behavior by influencing their internal cognitive systems [28]. Perceived personal rejection is a key factor that influences the internal cognitive systems of left-behind children and may be a potential mediator between peer attachment and pathological Internet use.

Perceived personal rejection is a subjective experience relating to an individual’s negative emotional experience that results from denial, indifference, and neglect by important others, such as parents, teachers, and peers. Rogers [29] suggested that acceptance and unconditional positive attention are foundational for mental health, and rejection is the basis of psychological disorders. Compared with objective rejection, perceived personal rejection is a psychological reality, which affects an individual’s psychology and behavior as an actual variable [27]. The association between rejection and a psychological disorder depends on how the rejection behavior is perceived [30]. The interpersonal risk model [31] posits that insecure peer attachment and other negative relational experiences with important others can lead to a series of cognitive and emotional problems that include rejection and depression. Acceptance–rejection theory [32] also emphasizes the influence of relationships with important others on children’s perceptions of rejection, positing that children’s experiences in their relationships with their parents and other attachment figures can strengthen their perception of parents’ acceptance and reduce that of rejection. Further, children’s relationships with and attachment to their peers are among their most important relationships and can be a predictor of perceived rejection.

According to acceptance–rejection theory, early experiences of being accepted and rejected have a lasting impact on children’s growth [32]. When children’s care needs and acceptance are not met, that is, when they perceive rejection by important others, a series of psychological and behavioral problems arise [33]. For example, a perception of rejection can make children hesitant or aggressive and hostile when interacting with others [34]. Adumitroaie and Dafinoiu [35] found that rejected teenagers are more aggressive than other teenagers and have lower self-esteem and a negative worldview. The self-medication theory suggests that PIU may be an adaptation to negative experiences and a coping strategy for reducing associated negative emotions [36]. Negative perceptions and emotions linked to reality prompt an individual to seek comfort and spend time in the virtual world of the Internet, which can lead to Internet addiction [2]. Empirical studies have also provided evidence in support of the above conclusion. For example, one study suggested that perceived rejection can predict the PIU of left-behind children [11]. Another study found that perceptions of rejection can lead to PIU among teenagers [37].

Therefore, three further hypotheses are proposed as follows:

**Hypothesis** **2** **(H2).** *Peer attachment can negatively predict perceived personal rejection among left-behind children.*

**Hypothesis** **3** **(H3).** *Perceived personal rejection has a positive effect on left-behind children’s pathological Internet use.*

**Hypothesis** **4** **(H4).** *Perceived personal rejection plays a mediating role in the relationship between left-behind children’s peer attachment and their pathological Internet use.*

### 1.3. Emotional Intelligence as a Moderator

Evidently, not all left-behind children who lack positive peer attachment will inevitably develop a perception of rejection that leads to PIU [11,22]. Left-behind children whose interpersonal environment is unfavorable may nevertheless develop healthy cognition and behavior, depending on whether protective factors are present, enabling them to resist the negative impacts of the unfavorable environment [38]. Emotional intelligence, which is often regarded as one of the best options for the discovery of protective factors, may be a protective factor that contributes to reducing the risk of insecure attachment and improving the positive effect of peer attachment [39]. Emotional intelligence refers to the ability of an individual to recognize, use, understand, and control emotional information and is considered an important protective resource [40].

Emotional intelligence may alleviate the impact of unsafe peer attachment on perceived rejection and PIU. The organism–environment interaction model [41] indicates that individuals develop in different ways given their intrinsic attributes. Therefore, even when they are in similar environments, they respond differently (are more or less sensitive), demonstrating different ways of adapting. These adaptations depend on the extent of the protective resources available to an individual. Resource protection theory posits that stress consumes individual resources and leads to negative outcomes, but that this negative impact can be alleviated if individuals have sufficient personal resources at their disposal [42]. The findings of empirical studies indicate that emotional intelligence can alleviate the risk effect of an individual’s insecure peer relationships on their perception of psychological pain and reduce their negative cognition [43].

Emotional intelligence can also play a protective role in preventing psychological and behavioral problems among left-behind children. It can help individuals to develop positive cognition and closer interpersonal relationships, thus eliminating the need for left-behind children to find interpersonal comfort and support in a virtual, Internet-based world [20]. According to the **protective–protective model** [44], when a protective factor exists (e.g., emotional intelligence), it will enhance the effect of another protective factor (e.g., peer attachment) [45] and reduce the risk of a negative behavior. Empirical studies have also shown that emotional intelligence can reduce the risk of PIU [43]. Moreover, some studies suggest that emotional balance is not only a negative predictor of individual problem behaviors (such as an addiction to Internet), but it is also a negative predictor of problem behaviors via other variables, for example, mental strength [46].

Accordingly, hypotheses 5 and 6 are presented as follows:

**Hypothesis** **5** **(H5).** *Emotional intelligence has a moderating effect on the relationship between peer attachment and perceived personal rejection.*

**Hypothesis** **6** **(H6).** *Emotional intelligence has a moderating role on the relationship between peer attachment and pathological Internet use.*

**Figure 1:**
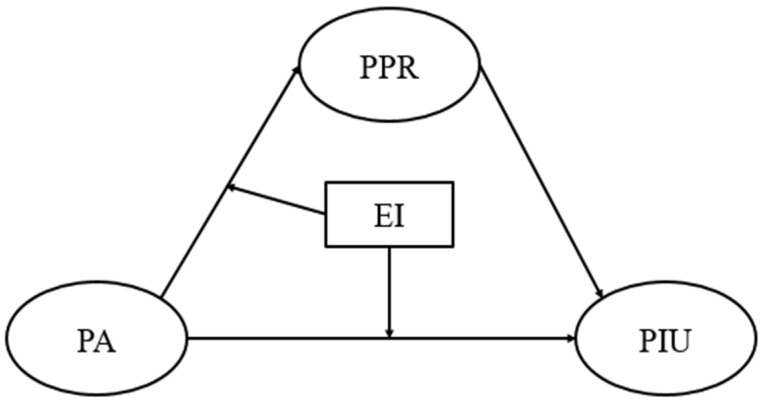
Research model. PA = peer attachment, PPR = perceived personal rejection, EI = emotional intelligence, PIU = pathological internet use.

The research framework is shown in Figure 1.

## 2. Materials and Methods

### 2.1. Sampling

This study was approved by the Zhejiang University ethics committee. A total of 406 left-behind children from six rural primary and secondary schools (three primary schools and three secondary schools) were surveyed using the cluster random sampling method. Two rural schools (one primary school and one secondary school) were located in eastern regions of China, two in central regions of China, and the remaining two in western regions of China. Before beginning the survey, we explained the purpose of our research to the school authorities and obtained their consent. We also explained the purpose of the study and the principles of confidentiality to the children to whom we distributed the questionnaire and obtained the participants and their parents’ consent. The children were informed that their participation was voluntary and that there would be no negative effects if they refused to participate or withdrew their participation.

Of all the children who agreed to participate, 202 (49.8%) were male and 204 (50.2%) were female. A total of 169 children (41.6%) were in elementary school, and 219 (53.9%) were in secondary school. The remaining 18 children (4.4%) did not report their stage of education. Of the total sample, 367 children (90.4%) were of Han ethnicity, and 39 children (9.6%) were from ethnic minority groups. A total of 206 (52.0%) left-behind children were boarding on campus, 189 (47.7%) were commuting between their homes and their schools, and 11 children (2.7%) did not indicate whether they were boarding or commuting. A total of 194 children (42.7%) reported that the economic status of their families was very poor or poor, 49.5% of the children reported a medium-level family economic status, and only 6.1% reported a rich or very rich family economic status. Eight of the left-behind children (1.8%) did not report their families’ economic status. The children’s average age was 12.76 ± 2.13 years, with their ages ranging between 8 and 16 years old.

### 2.2. Measures

Peer attachment. We applied the Peer Attachment Questionnaire obtained from the Inventory of Parent and Peer Attachment (IPPA), compiled by Armsden and Greenberg [47] and based on the theory of attachment, to assess the quality of children’s peer attachment. This questionnaire was subsequently revised by Wang et al. [48]. The scale comprises 25 items and is scored using a five-point Likert scale. Its coefficients were above 0.897, confirming its reliability. The scale included three dimensions named peer alienation, peer communication, and peer trust, and the alpha coefficients of the reliability indexes of the sub attachment scales were 0.87, 0.89, and 0.92, respectively.

Perceived personal rejection. The perceived rejection questionnaire was revised from the acceptance rejection questionnaire [32]. The questionnaire contains 9 items. Exploratory factor analysis (EFA) revealed that three factors (parent rejection, teacher rejection and peer rejection) were extracted through principal component analysis, and they explained 83.23% of the total variance. Confirmatory factor analysis showed that the fit index of χ^2^/df = 3.47 (<5), RMSEA = 0.074 (<0.08), CFI = 0.959 (>0.9) and TLI = 0.958 (>0.9) demonstrated a good fit with the three-factor model [49]. The Cronbach’s alpha coefficient for perceived personal rejection was 0.949, indicating a high level of reliability. All the above analyses confirmed that the validity and reliability of this scale were acceptable and satisfied the measuring requirements.

Emotional Intelligence. We used Wang’s [50] modified emotional intelligence scale to test left-behind children’s emotional intelligence. This scale was developed by Schutte et al. [51] and was subsequently revised and adapted to the Chinese cultural context by Wang [50]. The scale comprises 33 items and covers four dimensions, notably emotional self-cognition, self-management, others’ emotional management, and emotional application. It is scored using a five-point Likert scale. The scale demonstrated a high level of internal consistency and reliability (0.90) and retest reliability (0.78).

Pathological Internet use [52]. We used the pathological internet use scale (PIUS) developed by Yang and Lei [52]. This scale comprises 38 items and covers the following six dimensions: primacy, tolerance, compulsive Internet addiction treatment symptom, mood changes, comfort in social settings, and negative consequences. It is scored with a five-point Likert scale and has strong construct validity and reliability. The fit index was found to be satisfactory (χ^2^/df = 1.21, NFI = 0.90, NNFI = 0.97, CFI = 0.97, RMSEA = 0.03), the alpha coefficients for each of the above dimensions were 0.86, 0.77, 0.81, 0.87, 0.92, and 0.88, respectively, and the alpha coefficient for the total questionnaire was 0.94.

Demographic variables. We controlled the demographic variables, such as sex, grade, ethnicity, and age. Sex included male and female (e.g., 1 is for male and 2 is for female). Grade included primary school and junior middle school (e.g., 1 is for primary school students and 2 is for junior middle students). Ethnic was mainly divided into Han and ethnic minorities (e.g., 1 is affiliated with the Han group, 2 is affiliated with the minority group). In China, all the other 55 ethnic groups except the Han ethnicity are called ethnic minorities. Age was presented by asking the children to report how old they were.

### 2.3. Research Process

We first established a theoretical framework for the study and constructed a moderated mediation model. Subsequently, we selected a sample comprising 406 left-behind children from a total of six rural primary and secondary schools in mainland China. To ensure that the research was ethical, we obtained the consent of the school leaders, children, and their parents when conducting the survey, emphasizing that completion of the questionnaires was voluntary and ensuring the anonymity and confidentiality of the information collected. The third step in the research process entailed inputting the collected data and filtering out invalid data. The SPSS 21.0 software programs were used to perform the common method deviation test, descriptive statistical analysis of variables, and correlation analysis. The AMOS 21.0 software program was used to perform the common method deviation test and the moderated mediation model.

## 3. Results

### 3.1. Common Method Bias Test

Common method bias was tested using Harman’s one-factor test [53]. Variables such as peer attachment, perceived personal rejection, emotional intelligence, and PIU are typically assessed using EFA, which is performed to test the size of the interpretation ratio of the first common factor in unrotated factor analyses [21]. The EFA results in this study showed that the interpretation ratio of the first common factor was 27.43%, which was below 40%. We further adapted confirmatory factor analysis and set the common factor number as 1. These results demonstrated an unsatisfactory fit index (χ^2^/df = 17.438 > 5, RMSEA = 0.082 > 0.07, NFI = 0.89 < 0.9, TLI = 0.88 < 0.9, CFI = 0.87 < 0.9). Both of the above methods confirmed that there was no serious common method bias. Therefore, further analysis could be conducted.

### 3.2. Correlations

Table 1 indicated that the univariate correlation between sex (1 is for male and 2 is for female) and perceived personal rejection was significant (r = −0.117, *p* < 0.05), as was the correlation between sex and PIU (r = −0.174, *p* < 0.01). Females have low level of perceived personal rejection and PIU than males. No significant correlations were found between sex and peer attachment (r = 0.057, *p* > 0.05) or between sex and emotional intelligence (r = 0.001, *p* > 0.05). There were significant correlations between grade and peer attachment (r = 0.145, *p* < 0.01), grade and perceived personal rejection (r = 0.164, *p* < 0.01), grade and PIU (r = 0.157, *p* < 0.01), and grade and emotional intelligence (r = 0.103, *p* < 0.05). Junior middle students have higher peer attachment, perceived personal rejection, PIU, and emotional intelligence than primary school students. The univariate correlation between age and peer attachment was significant (r = 0.145, *p* < 0.01), as it was between age and perceived personal rejection (r = 0.164, *p* < 0.05), between age and emotional intelligence (r = −0.103, *p* < 0.05), and between age and PIU (r = −0.157, *p* < 0.01). No significant correlations were found between ethnicity (1 is affiliated with the Han group, 2 is affiliated with the minority group) and peer attachment (r = 0.069, *p* > 0.05), between ethnicity and perceived personal rejection (r = −0.019, *p* > 0.05), between ethnicity and emotional intelligence (r = 0.050, *p* > 0.05), or between ethnicity and PIU (r = −0.024, *p* > 0.05).

The univariate correlation between peer attachment and perceived personal rejection (r = −0.188, *p* < 0.01) was negative, as it was between peer attachment and PIU (r = −0.186, *p* < 0.01). There were positive correlations between peer attachment and emotional intelligence (r = 0.402, *p* < 0.01) and between perceived personal rejection and PIU (r = 0.575, *p* < 0.01). Emotional intelligence was negatively correlated with perceived personal rejection (r = −0.261, *p* < 0.01) and with PIU (r = −0.171, *p* < 0.01).

### 3.3. A Moderated Mediating Effect Model

**Figure 2:**
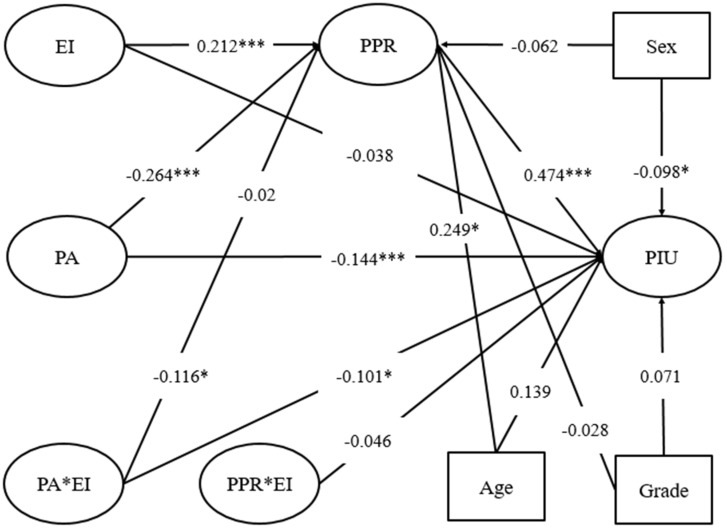
The mediating effect of perceived personal rejection on the relationship between peer attachment and pathological Internet use. PA = peer attachment, PPR = perceived personal rejection, EI = emotional intelligence, PIU = pathological Internet use. * *p* < 0.05, *** *p* < 0.001.

AMOS version 21.0 was used to construct the structural equation model (Figure 2), and a good fit was found between the model and the actual data (χ^2^/df = 2.386 < 5; RMSEA = 0.058 < 0.07; GFI = 0.902 > 0.90; NFI = 0.908 > 0.90; TLI = 0.929 > 0.90; CFI = 0.944 > 0.90). After the effects of sex and grade had been controlled for, peer attachment negatively predicted perceived personal rejection (β = −0.264, *p* < 0.001), while perceived personal rejection positively predicted PIU (β = 0.474, *p* < 0.001). Peer attachment still had a significantly negative effect on PIU (β = −0.144, *p* < 0.001), indicating that perceived personal rejection had a partial mediating role in the relationship between peer attachment and PIU.

**Figure 3:**
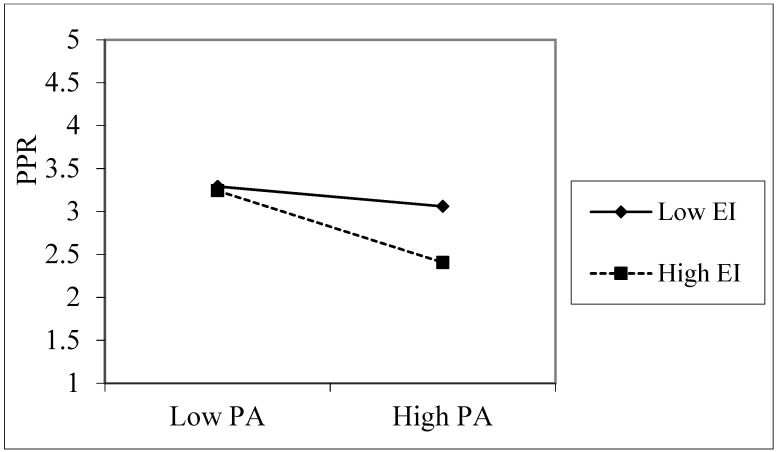
The interaction effect of peer attachment with emotional intelligence on perceived personal rejection. PA = peer attachment, PPR = perceived personal rejection, EI = emotional intelligence, PIU = pathological Internet use.

Emotional intelligence had a positive effect on perceived personal rejection (β = 0.212, *p* < 0.001), while the interaction terms of peer attachment and emotional intelligence had a negative effect on perceived personal rejection (β = −0.116, *p* < 0.05). Thus, emotional intelligence moderated the relationship between peer attachment and perceived personal rejection. To test the moderating effect of personal intelligence on the impact of peer attachment on perceived personal rejection further, we constructed an interactive effect map (Figure 3). The results of a simple slope test [27] indicated that, for children with a low level of emotional intelligence (e.g., emotional intelligence = −1 SD), perceived personal rejection showed a significant downward trend (γ = −0.11, t = −3.03, *p* < 0.05) with an increase in peer attachment. However, for children with a high level of emotional intelligence (e.g., emotional intelligence = +1 SD), perceived personal rejection showed a more significant downward trend (γ = −0.23, t = −4.56, *p* < 0.01) with an increase in peer attachment. These results suggest that peer attachment can facilitate a reduction in perceptions of personal rejection among left-behind children, especially when their level of emotional intelligence is high.

**Figure 4:**
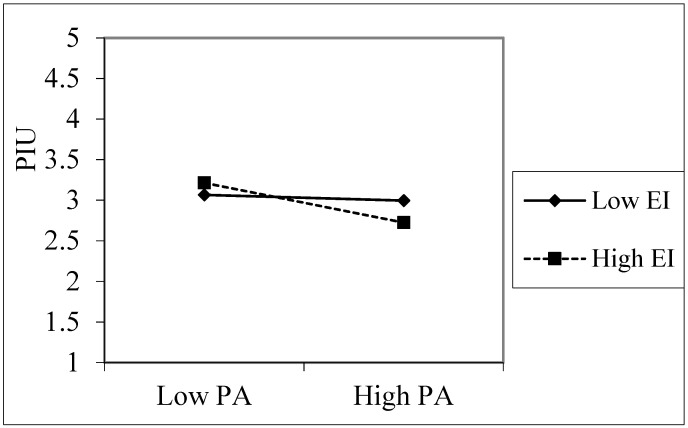
The interaction effect of peer attachment with emotional intelligence on pathological Internet use. PA = peer attachment, PPR = perceived personal rejection, EI = emotional intelligence, PIU = pathological Internet use.

The interaction terms for peer attachment and emotional intelligence also had a negative impact on PIU (β = −0.101, *p* < 0.05), indicating that emotional intelligence has a moderating effect on the relationship between peer attachment and perceived personal rejection (Figure 4). The results of a simple slope test showed that, for children with a low level of emotional intelligence (e.g., emotional intelligence = −1 SD), PIU did not show a significant change with an increase in peer attachment. However, for children with a high level of emotional intelligence (e.g., emotional intelligence = +1 SD), PIU showed a significant downward trend (γ = −0.21, t = −3.56, *p* < 0.05) with an increase in peer attachment.

## 4. Discussion

We explored the effects of peer attachment on left-behind children’s PIU. Specifically, we examined the mediating effect of perceived personal rejection on the relationship between peer attachment and PIU. We further examined the moderating effect of emotional intelligence on the relationship between peer attachment and perceived personal rejection and between peer attachment and PIU. Our findings were consistent with the theory of developmental psychopathology [14]. Moreover, they contribute empirically to advancing understanding on the role of peer attachment, perceived personal rejection, and emotional intelligence in the development of left-behind children’s PIU.

Peer attachment can significantly predict left-behind children’s PIU. This conclusion is consistent with that of the previous studies [22,23,24]. Lower level of peer attachment is an important risk environmental factor according to the theory of developmental psychopathology [14], and it can correspond to a higher risk of adolescents developing an addiction to the Internet [22]. That is because children who face developmental risk factors lack an “arena of comfort” in real life and are likely to seek compensation by turning to the virtual world of the Internet. One study posits that, in cases of adolescents’ impeded mental development, the Internet has a compensatory function [54]. Left-behind children with low levels of peer attachment tend to seek to escape from the real world and form compensatory interpersonal relationships on the Internet that provide them with emotional comfort. However, this behavior may lead to the development of an addiction to the Internet [55]. Moreover, another study suggests that adolescents with low levels of peer attachment who overuse the Internet may do so because their need to belong is not met within their existing social relationships, leaving them frustrated [56]. Teenagers with unmet needs relating to belonging are more likely to be addicted to the Internet [57].

Our results indicate that perceived personal rejection has a partial mediating effect on the relationship between peer attachment and PIU. Thus, peer attachment can contribute partially to a reduction in the PIU of left-behind children by reducing their perceived personal rejection. Left-behind children use inappropriate psychological compensation methods (e.g., online games) to satisfy their developmental needs, such as forming close relationships with their peers, and further reduce their negative cognition of the environment. However, when their needs are not satisfied, a process of “pathological compensation” relating to Internet use occurs [54]. Studies indicate that social isolation and psychological maladjustment caused by social isolation are key factors relating to PIU [2]. Perceived personal rejection is a kind of psychological maladjustment, which is the most critical and stable proximal factor that affects the establishment and maintenance of PIU. The individual’s relationship with their environment (e.g., with their peers) is a distal risk factor affecting PIU through the mediating effect of a proximal factor [58]. In addition, relationship needs are basic components of individual needs and serve as essential “nutrients” fostering individual happiness and healthy growth. If such needs (e.g., peer attachment) are not met in real life, negative cognition (e.g., perceptions of rejection) correspondingly increases, inducing problematic behavior, such as PIU [59].

Emotional intelligence plays a moderating role in the relationship between peer attachment and perceived personal rejection and between peer attachment and PIU. Peer attachment has a more negative impact on perceived personal rejection and the PIU of left-behind children with high levels of emotional intelligence than it does on left-behind children with low levels of emotional intelligence. This result supports the protective-protective model posits that one high-level protective factor can significantly enhance the function of another protective factor [44,60]. As an important protective factor, emotional intelligence can enhance the role of peer attachment in reducing left-behind children’s perceptions of personal rejection and their PIU. However, lower levels of emotional intelligence are associated with lower levels of efficacy of peer attachment in reducing perceptions of personal rejection among left-behind children and their PIU.

From another perspective, this result supported the importance of resilience emphasized by the theory of developmental psychopathology [14]. Some individuals from disadvantaged backgrounds with high protection factors may still be able to adapt to the environment. Left-behind children are more likely to experience unsafe peer relationships and even peer aggression [22]. Those children with higher levels of emotional intelligence can better buffer these negative external effects and prevent further negative cognition. Emotional intelligence can help to promote an individual’s positive emotional experience, which contributes to improving adaptation. In the short term, enhanced attention, cognition, and action responses can lead to the construction of psychological resources and the reduction in negative cognition [61], all of which can contribute to a reduced risk of PIU.

This study had some limitations. First, we used a self-reporting method to test peer attachment, perceived personal rejection, emotional intelligence, and PIU. In the future, more diversified measurements, such as reports obtained from partners, teachers, and parents should be applied. Second, the relationships among the variables were determined using a cross-sectional research method. In future studies, the application of a longitudinal research method should be further explored to provide more evidence for the relationships among the variables. Third, perceived personal rejection only played a partial mediating role on the relationship between peer attachment and PIU. Consequently, more mediating variables should be used in future studies to determine the impact mechanism of peer attachment on PIU. Fourth, the type of attachment of left behind children to their parents was not controlled in this study, and the moderated mediating effect model was not compared between left behind and non-left behind children. In future research, more relevant variables will be reasonably controlled, and the relationship in the model will be better compared between different groups.

## 5. Conclusions

Notwithstanding the above limitations, to the best of our knowledge, this is the first study to examine the relationships between peer attachment, perceived personal rejection, emotional intelligence, and PIU. Our study is the first to explore the influence mechanism of peer attachment relating to PIU from the perspective of perceived rejection. Our findings confirmed that peer attachment is a protective factor, which can negatively impact on their PIU partially by reducing their perceptions of personal rejection. Emotional intelligence was found to be a stable moderator, which can improve the protective role of peer attachment in reducing the risk of left-behind children’s perceptions of rejection and their PIU.

This study highlights the role of peer attachment as a protective factor that should be accorded more attention in efforts to reduce PIU among left-behind children. Further, effective interventions are required to reduce perceived personal rejection, which was found to be not only a mediating factor between peer attachment and PIU but also a key proximal factor leading to pathological Internet use. In addition, training interventions should be implemented to enhance left-behind children’s emotional intelligence, with the aim of reducing their risk of developing an addiction to the Internet.

## Figures and Tables

**Table 1:** Correlations.

	Sex	Grade	Age	Ethnic	Peer Attachment	Perceived Personal Rejection	Emotional Intelligence	Pathological Internet Use
Sex	1							
Grade	−0.089	1						
Age	−0.075	0.898 **	1					
Ethnic	−0.043	0.288 **	0.288 **	1				
Peer attachment	0.057	0.145 **	0.145 **	0.069	1			
Perceived personal rejection	−0.117 *	0.164 **	0.164 **	−0.019	−0.188 **	1		
Emotional intelligence	0.001	0.103 *	0.103 *	0.050	0.402 **	−0.261 **	1	
Pathological Internet use	−0.174 **	0.157 **	0.157 **	−0.024	−0.186 **	0.575 **	−0.171 **	1

* *p* < 0.05, ** *p* < 0.01.

## Data Availability

Data is available from the corresponding author upon reasonable request.
